# Prevalence and influencing factors of malnutrition in diabetic patients: A systematic review and meta‐analysis

**DOI:** 10.1111/1753-0407.13610

**Published:** 2024-10-04

**Authors:** Tong Zhang, Jiangxia Qin, Jiali Guo, Jianhui Dong, Junbo Chen, Yuxia Ma, Lin Han

**Affiliations:** ^1^ Evidence‐Based Nursing, School of Nursing Lanzhou University Lanzhou China; ^2^ Department of Nursing Gansu Provincial Hospital Lanzhou China

**Keywords:** diabetes, influencing factors, malnutrition, meta‐analysis, prevalence

## Abstract

The prevalence of malnutrition in diabetic patients and its influencing factors remain poorly described. We aim to investigate the prevalence of malnutrition and the influencing factors in diabetic patients through meta‐analysis. Utilizing search terms, such as diabetes, malnutrition, and prevalence, we systematically searched eight databases, including Embase, PubMed, Web of Science, The Cochrane Library, China Knowledge Resource Integrated Database (CNKI), Wanfang Database, Chinese Biomedical Database (CBM), and VIP Database, from inception to May 4, 2023. The search aimed to identify studies related to the prevalence of malnutrition and its influencing factors in adult patients with diabetes. Cohort studies, case–control studies, and cross‐sectional studies that met the inclusion criteria were included in the analysis. Stata 16.0 software was used for meta‐analysis. Quality of the evidence was assessed using Grading of Recommendations, Assessment, Development and Evaluation (GRADE). The study protocol is registered with Prospective Register of Systematic Reviews (PROSPERO), CRD42023443649. A total of 46 studies were included, involving 18 062 patients with ages ranging from 18 to 95 years. The overall malnutrition prevalence was 33% (95% confidence interval [CI]: 0.25–0.40), compared with an at‐risk prevalence of 44% (95% CI: 0.34–0.54). Sixteen factors associated with malnutrition in diabetic patients were identified. This meta‐analysis provides insights into the prevalence of malnutrition and its risk factors in diabetic patients. Regular nutritional screening for patients with risk factors is essential for early detection and intervention.

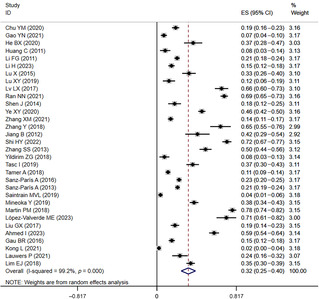

## Graphical abstract

Highlights

- The overall prevalence of malnutrition in diabetic patients was 33% (95% CI: 0.25–0.40), and the at‐risk prevalence of malnutrition was 44% (95% CI: 0.34–0.54).
- Diabetic patients with malnutrition had significantly lower levels of body mass index, albumin, hemoglobin, triglyceride, and high‐density lipoprotein cholesterol, while showing an increase in glycosylated hemoglobin.
- C‐reactive protein >10 mg/L, age, duration of diabetes, Wagner grades 3–5, with cardiovascular disease, and using insulin were identified as risk factors for malnutrition in diabetic patients.

## 1 INTRODUCTION

Diabetes mellitus (DM) is a prevalent and potentially devastating disease, with its prevalence on the rise in recent decades, presenting a significant public health challenge in the 21st century.^1^ Chronic complications traditionally associated with diabetes include diabetic macroangiopathy, diabetic peripheral neuropathy, diabetic retinopathy, diabetic nephropathy, and diabetic foot.^2^ However, nutrition‐related comorbidities in diabetes, especially malnutrition, have often been neglected, despite their significant impact on various body systems and potentially fatal consequences.^3^, ^4^, ^5^ These patients face a higher risk of complications, prolonged hospitalization, and mortality.

Malnutrition refers to a state in which physiological and psychological functions are impaired due to inadequate nutrient intake or excessive expenditure, leading to compromised clinical outcomes of diseases.^6^ Concurrent malnutrition is a crucial factor contributing to the deterioration of diabetic patients' prognosis, especially among the elderly who are more susceptible to disease‐related malnutrition.^6^, ^7^ Some people develop malnutrition because there is not enough food available, or because they have difficulty eating or absorbing nutrients, or because digestive or stomach diseases prevent the digestion and absorption of nutrients, or because they deliberately reduce their food intake in order to reduce their body mass index, etc. However, the causes of malnutrition in diabetic patients may be related to the pathophysiological mechanisms of diabetes, such as persistent hyperglycemia, chronic inflammation, and insulin resistance. Diabetic patients, being in a state of chronic inflammation, experience increased protein catabolism. Persistent hyperglycemia and insulin resistance can impair digestive system function, hindering nutrient digestion and absorption,^8^ thereby promoting malnutrition. Additionally, diabetic patients often face dietary restrictions, especially regarding carbohydrate intake. This condition promotes oxidative stress, heightens the risk of chronic complications, and worsens nutritional status. Furthermore, the use of nutrient‐restricted diets contributes to an unbalanced energy balance.^9^ As a result, malnutrition can trap diabetics in a vicious cycle, underscoring the urgent need to comprehend the nutritional status of diabetics and the factors influencing malnutrition.

However, the nutritional status of diabetics are substantial discrepancies among different studies, especially the prevalence of malnutrition in diabetics ranging from 2%^10^ to 78%.^11^ Underestimating the prevalence of malnutrition may reduce the importance of malnutrition in clinical practice, with adverse consequences. Based on current nutritional assessment tools, the prevalence of malnutrition and the prevalence of at‐risk for malnutrition can be used as the best evidence‐based basis for estimating nutritional status in patients with diabetes. Various studies reported the influencing factors related to malnutrition in diabetics, including body mass index (BMI),^4^, ^12^, ^13^, ^14^, ^15^, ^16^, ^17^, ^18^ albumin (ALB),^3^, ^4^, ^12^, ^17^, ^19^, ^20^ triglyceride (TG),^12^, ^21^, ^22^, ^23^ C‐reactive protein (CRP),^24^, ^25^, ^26^ >10 mg/L, age,^3^, ^4^, ^17^, ^20^, ^23^ duration of diabetes,^20^, ^23^ smoking,^18^, ^25^ gender,^27^, ^28^, ^29^ lower education level,^29^, ^30^ and others. However, some of these reports present controversial findings and lack sufficient credibility. Therefore, the objective of this study was to conduct a systematic review and meta‐analysis to summarize the prevalence of malnutrition and the prevalence of at‐risk for malnutrition in diabetic patients. Additionally, we aim to analyze the influencing factors associated with malnutrition and provide a basis for early nutritional intervention in diabetics, so as to improve the quality of life of diabetics and reduce the burden on families and society.

## 2 MATERIALS AND METHODS

### 2.1 Protocol and registration

This meta‐analysis was performed according to the Preferred Reporting Items for Systematic Reviews and Meta‐Analyses (PRISMA) guidelines and the study protocol was registered in the International Prospective Register of Systematic Reviews (PROSPERO) [CRD42023443649].

### 2.2 Search strategy

In this review, a comprehensive search was conducted across eight databases, including Embase, PubMed, Web of Science, The Cochrane Library, China Knowledge Resource Integrated Database (CNKI), Wanfang Database, Chinese Biomedical Database (CBM), and VIP Database (VIP), from their inception until May 4, 2023. Additionally, manual searches of reference citations were performed to ensure the comprehensiveness of the retrieved studies. The search terms were developed using a combination of free terms and Mesh terms, and the Boolean operators OR/AND were used for their combination. Detailed search strategies employed in all databases can be found in Table S1.

### 2.3 Selection criteria

Inclusion criteria included the following: (1) The study design was either a cohort study, case–control study or cross‐sectional study. (2) All participants were diabetic patients aged 18 years and over, with or without chronic complications of diabetes, and their race and nationality were not limited. (3) The exposure factor was malnutrition or at‐risk for malnutrition. (4) The study reported the prevalence of malnutrition or at‐risk for malnutrition and influencing factors of malnutrition in diabetic patients.

Exclusion criteria included the following: (1) Studies that diagnosed malnutrition solely based on low BMI, as this may compromise the accuracy of malnutrition diagnosis.^31^ (2) Studies with a sample size smaller than 50. (3) Studies with missing data that could not be obtained by contacting the author. (4) The study which participants were on peritoneal dialysis or were complicated with diseases such as cancer and tuberculosis that severely affected nutritional status. (5) Review articles, meta‐analyses, letters, conference abstracts, animal experimental studies, and other nonrelevant article types.

### 2.4 Study screening and data extraction

Two researchers (Zhang, T., and Qin J.) independently extracted data from the included literature after the screening process. The extracted data included the first author's name, publication year, study region, study type, definition of malnutrition, sample size, age, gender, and influencing factors of malnutrition. In cases of disagreements, a third investigator (Ma, Y.) was available for discussion and resolution.

### 2.5 Quality assessment

Two researchers (Zhang, T, and Qin J.) independently used the Joanna Briggs Institute (JBI) critical appraisal checklist for cross‐sectional studies,^32^ studies that had 50% or more ‘Yes’ across the quality assessment parameters were considered low risk and the Newcastle‐Ottawa Scale (NOS) for cohort studies and case–control studies.^32^ This scale is divided into three categories (selection, comparability, and exposure), with a total of eight items for study population selection (four items), comparability (one item), and exposure or outcome evaluation (three items), with a maximum score of 9. Studies with a score ≥6 were considered as the high quality. Any conflict was resolved by discussion with the third investigator (Ma, Y.).

### 2.6 Statistical analysis and quality of the evidence

The pooled prevalence of malnutrition or the pooled prevalence of at‐risk for malnutrition was evaluated based on the nutrition measurement tools used in the studies included in this meta‐analysis, such as the Miniature Nutrition Assessment Scale (MNA), where a score of <17.0 indicates malnutrition, a score ranging from 17.0 to 23.9 indicates at‐risk for malnutrition, and a score of ≥24.0 indicates a favorable nutritional status. To avoid duplication, estimates from distinct studies employing multiple measurement tools to assess nutritional status were amalgamated based on their respective primary diagnostic criteria.

Stata 16.0 software was utilized for conducting the meta‐analysis. Odds ratio (OR) was employed as the effect index for count data, while mean difference (MD) was used for measurement data. Each effect size was presented with a point estimate and a 95% confidence interval (CI), and statistical significance was set at *p* < 0.05. To assess heterogeneity among the included studies, the *Q* test with *α* = 0.1 was performed, and the magnitude of heterogeneity was quantified using *I* ^*2*^. If *p* ≥ 0.1 and *I* ^*2*^ ≤ 50%, heterogeneity was considered acceptable, and the fixed‐effect model was applied. On the other hand, if *I* ^*2*^ > 50% and *p* < 0.1, indicating significant heterogeneity, the random‐effects model was used. Additionally, subgroup analysis was conducted for categorical variables (measurement tool, country, and chronic complications of diabetes), chi‐square (*χ* ^*2*^) test was used to explore potential differences in prevalence across subgroups. Sensitivity analysis was performed by removing each study individually to assess the consistency and quality of the results regarding malnutrition prevalence and the prevalence of at‐risk for malnutrition. Publication bias was assessed using Egger's test^33^ for funnel plot asymmetry in addition to visual inspection of the funnel plots.

Finally, two researchers (Zhang, T, and Qin J.) used the Grading of Recommendations, Assessment, Development and Evaluation (GRADE) to assess the quality of evidence between influencing factors with malnutrition. According to the GRADE approach, we rated the quality as four levels: high, moderate, low, and very low.

## 3 RESULTS

### 3.1 Study selection

**FIGURE 1:**
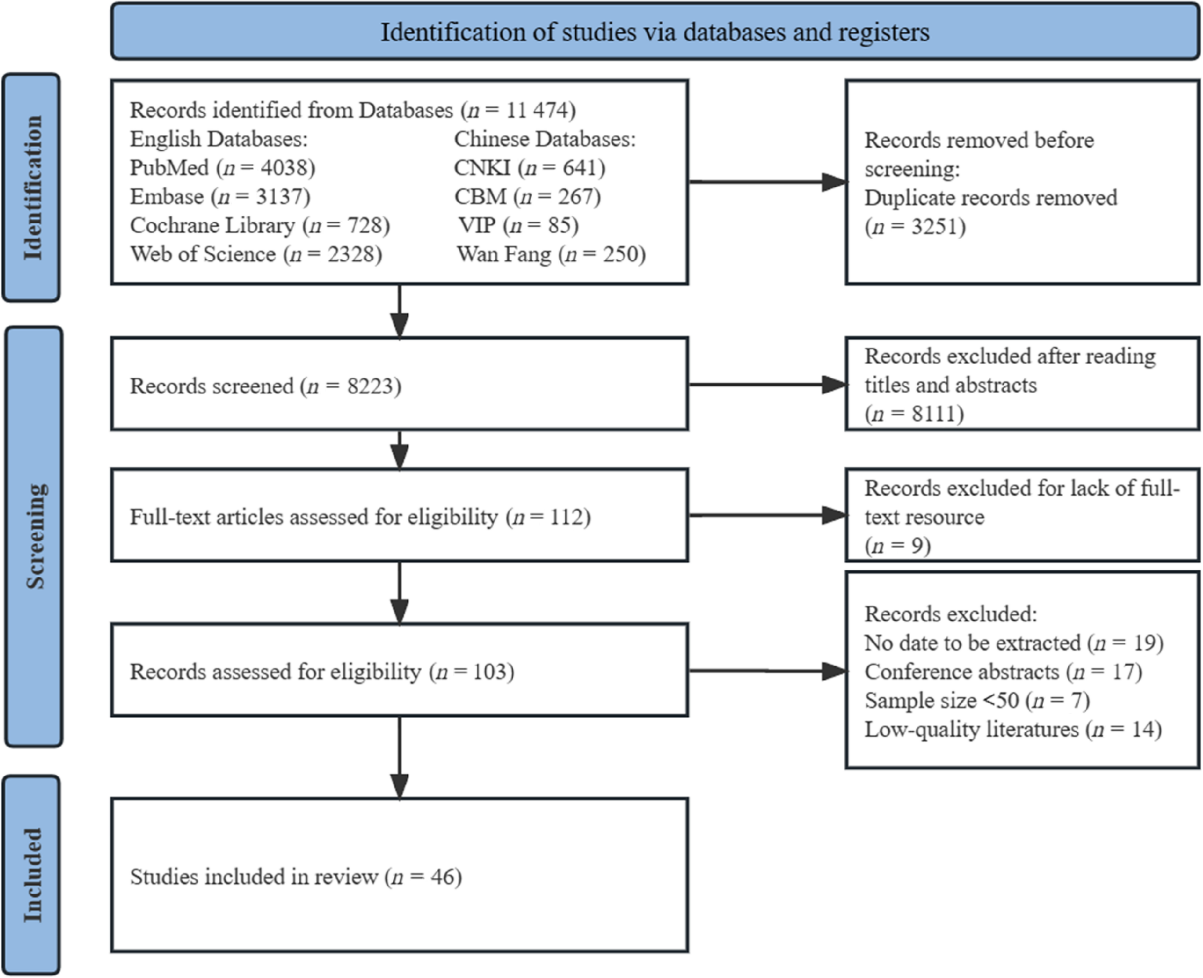
The flow chart of studies selecting process. CBM, Chinese Biomedical Database; CNKI, China Knowledge Resource Integrated Database.

The initial search retrieved 11 474 articles, of which 3251 were duplicates. After screening titles and abstracts, there are 112 articles remaining. These articles were further read in full text and evaluated in detail. Eventually, 46 articles were included in the meta‐analysis. The literature search and screening procedures are shown in Figure 1.

### 3.2 Baseline characteristics of the included studies

The main characteristics of the included studies are presented in Table 1. A total of 46 studies involving 18 062 patients were included. The included studies comprised 36 cross‐sectional studies,^8^, ^10^, ^12^, ^13^, ^15^, ^17^, ^19^, ^20^, ^21^, ^22^, ^23^, ^24^, ^25^, ^26^, ^28^, ^29^, ^30^, ^34^, ^35^, ^36^, ^37^, ^38^, ^39^, ^40^, ^41^, ^42^, ^43^, ^44^, ^45^, ^46^, ^47^, ^48^, ^49^, ^50^, ^51^, ^52^ and 10 cohort studies,^3^, ^4^, ^11^, ^14^, ^18^, ^27^, ^53^, ^54^, ^55^, ^56^ published between 2011 and 2023, with sample sizes ranging from 60^19^ to 1754.^54^ These studies were conducted in various countries, including China,^3^, ^8^, ^10^, ^12^, ^13^, ^18^, ^19^, ^21^, ^22^, ^23^, ^24^, ^25^, ^26^, ^28^, ^34^, ^35^, ^36^, ^37^, ^38^, ^39^, ^40^, ^41^, ^42^, ^44^, ^49^, ^50^, ^51^, ^52^, ^53^, ^56^ Turkey,^29^, ^46^, ^47^, ^48^ Spain,^4^, ^11^, ^27^, ^55^ Japan,^15^, ^17^, ^20^, ^54^ Brazil,^30^ Pakistan,^43^ Belgium,^14^ and Korea.^45^ Further details regarding the measurement tools used to assess nutritional status in the included studies can be found in Table S2.

**TABLE 1:** Characteristic of included studies.

Author (year)	Study country	Study design	Sample (*N*)	Mean age	Participants	Measurement tools of nutrition status	Prevalence (%)		Influencing factors
			T‐M‐F				Malnutrition^a^	Risk^b^	
fet al. (2023)	Pakistan	Cross‐sectional	359–153‐206	52.61 ± 11.80	T2DM	SGA	58.8	‐	14
Chen et al. (2023)	China	Cross‐sectional	90‐47‐43	≥60	DN	NRS‐2002	‐	62.2	1, 3, 4
Chu et al. (2020)	China	Cross‐sectional	426‐271‐155	≥65	T2DM	MNA‐SF	19.3	56.1	3, 4, 5
Gau et al. (2016)	China	Cross‐sectional	478‐271‐207	65.4 ± 13.1	DFU	MNA	15.2	85.4	NA
Gao et al. (2021)	China	Cross‐sectional	252‐126‐126	65–88	T2DM	MNA‐SF	7.1	56.8	1, 2, 3, 4, 5, 10
He et al. (2020)	China	Cross‐sectional	102‐0‐102	≥60	DFU	MNA‐SF	37.3	‐	7, 9, 15
Huang et al. (2011)	China	Cross‐sectional	106‐59‐47	35–86	DM	MNA	8.5	72.6	NA
Huang et al. (2020)	China	Cohort	319‐201‐118	56.36 ± 8.55	DFU	NRS‐2002	‐	39.5	8, 9
Huang et al. (2022)	China	Cross‐sectional	447‐227‐220	M:60.94 ± 11.54 F:64.94 ± 10.53	T2DM + macrovascular disease	NRS‐2002	‐	27.3	1, 3, 4, 10
Ji et al. (2022)	China	Cross‐sectional	610	66 (63,70)	T2DM	GNRI	‐	19.5	1, 2, 3, 4, 6, 10
Jiang et al. (2012)	China	Cross‐sectional	60‐33‐27	48–80	T2DM	SGA	41.7	‐	2, 6
Keskinler et al. (2021)	Turkey	Cross‐sectional	222‐90‐132	58.1 ± 10	T2DM	NRS‐2002	‐	14.4	2, 13
Kimura et al. (2021)	Japan	Cohort	1754‐1231‐523	75 (70,79)	T2DM	GNRI	‐	49.3	16
Kong et al. (2017)	China	Cross‐sectional	168‐93‐75	67–95	DN	NRS‐2002	‐	30.4	NA
Kong et al. (2021)	China	Cross‐sectional	291‐137‐154	69 (67,72)	T2DM	MNA	2.1	35.1	NA
Lauwers et al. (2021)	Belgium	Cohort	110‐88‐22	68 ± 12	DFU	GLIM	23.6	‐	1
Lai et al. (2022)	China	Cross‐sectional	603‐314‐289	62.66 ± 11.34	T2DM + chronic complications	NRS‐2002	‐	25.0	2
Li et al. (2011)	China	Cross‐sectional	775‐379‐396	NRI > 100: 62.1 ± 12.9 NRI ≤ 100: 61.5 ± 13.9	T2DM	NRI	20.8	‐	1, 3, 4, 5
Li et al. (2023)	China	Cross‐sectional	454‐284‐170	27–87	T2DM	CONUT	15.0	‐	1, 3, 5, 6.11, 12, 15
Liu et al. (2017)	China	Cohort	302‐194‐108	80 (72,84)	T2DM	MNA	18.5	51.7	2
Lim et al. (2018)	Korea	cross‐sectional	464‐0‐464	69.6 ± 2.96	DM	NSI	34.7	‐	NA
López‐Valverde et al. (2023)	Spain	Cohort	77‐57‐20	69.6	DFU	GLIM	71.4	‐	1, 2, 3
Lu et al. (2015)	China	Cross‐sectional	176	74.94 ± 8.83	DN	MNA	33.0	‐	NA
Lu et al. (2019)	China	Cross‐sectional	105‐64‐41	60–88	DM	MNA	12.4	71.4	NA
Lv et al. (2017)	China	Cross‐sectional	182‐102‐80	60–84	DFU	MNA	66.5	‐	NA
Martin et al. (2018)	Spain	Cohort	402‐179‐223	80.8 ± 8.5	T2DM	MNA	77.6	‐	NA
Mineoka et al. (2019)	Japan	Cross‐sectional	461‐272‐189	66.5 ± 12.9	T2DM	CONUT	38.2	‐	1, 6
Pan et al. (2016)	China	Cross‐sectional	456‐280‐176	18–90	T2DM	NRS‐2002	‐	21.5	NA
Ran et al. (2021)	China	Cross‐sectional	559‐321‐238	60–88	DFU	MNA	69.2	87.5	7, 9, 15
Saintrain et al. (2019)	Brazil	Cross‐sectional	246‐108‐138	65–94	DM	MNA	3.7	19.5	13, 14
Sanz‐París et al. (2016)	Spain	Cohort	1014‐504‐510	77.9 ± 6.92	T2DM	MNA‐SF	29.6	60.1	NA
Sanz‐París et al. (2013)	Spain	Cohort	1098‐548‐549	78 ± 7.1	DM	MNA	21.2	60.4	8
Shen et al. (2014)	China	Cross‐sectional	132‐68‐64	≥80	DM	MNA‐SF	18.2	62.9	8
Shi et al. (2019)	China	Cross‐sectional	512‐263‐249	27–89	T2DM	NRS‐2002	‐	18.0	4
Shi et al. (2022)	China	Cross‐sectional	357‐237‐120	62.35 ± 11.26	DFU	CONUT	72.0	0	8
Shiroma et al. (2023)	Japan	Cross‐sectional	479‐264‐215	71 (62, 77)	T2DM	GNRI	‐	23.2	2, 3, 6, 16
Takahash et al. (2021)	Japan	Cross‐sectional	526‐301‐225	67.1 ± 10.9	T2DM	GNRI	‐	5.1	1, 2, 3, 4
Tamer et al. (2018)	Turkey	Cross‐sectional	580‐253‐327	54.6 ± 10.7	T2DM	SGA	11.4	‐	NA
Tasc et al. (2019)	Turkey	Cross‐sectional	215‐64‐151	>65	T2DM	MNA‐SF	36.7	‐	8, 13, 14, 16
Xie et al. (2017)	China	Cohort	271‐162‐109	66.9 ± 11.1	DFA	GNRI	‐	50.9	1, 3, 5, 11, 12
Xu et al. (2014)	China	Cross‐sectional	178‐108‐70	M: 58.4 ± 12.9 F: 63.7 ± 10.4	DM	NRS‐2002	‐	29.8	NA
Ye et al. (2020)	China	Cross‐sectional	671	≥40	T2DM	CONUT	46.2	‐	4, 5, 6, 10, 12
Yildirim et al. (2018)	Turkey	Cross‐sectional	104‐67‐37	65.08 ± 12.57	T2DM	MNA	7.7	26.0	NA
Zhang et al. (2013)	China	Cohort	252‐155‐97	68.6 ± 11.3	DFU	SGA	50.0	‐	NA
Zhang et al. (2021)	China	Cross‐sectional	539‐307‐232	72.26 ± 16.39	T2DM	MNA	14.1	70.1	1, 4, 5, 10
Zhang et al. (2018)	China	Cross‐sectional	78‐41‐37	≥60	T2DM	MNA	65.4	‐	NA

*Note*: Participants: diabetic nephropathy (DN); type 2 diabetes mellitus (T2DM); diabetic foot ulcers (DFU); diabetic foot amputation (DFA); diabetes mellitus (DM); diabetes chronic diseases including diabetic macrovascular disease, diabetic peripheral neuropathy, diabetic retinopathy, diabetic nephropathy and diabetic foot. Measurement tools of nutrition status: Mini Nutritional Assessment (MNA); Short‐Form Mini Nutritional Assessment (MNA‐SF); Subjective Global Assessment (SGA); Nutritional Risk Index (NRI); Global Leadership Initiative on Malnutrition (GLIM); Controlling Nutritional Status (CONUT); Nutritional Screening Initiative (NSI); Nutritional Risk Screening 2002 (NRS‐2002); Geriatric Nutritional Risk Index (GNRI). Influencing factors: (1) body mass index (BMI); (2) age; (3) albumin (ALB); (4) glycosylated hemoglobin (HbA1c); (5) hemoglobin (Hb); (6) duration of diabetes; (7) diabetic foot infection; (8) gender; (9) Wagner grades 3–5; (10) triglyceride (TG); (11) smoking; (12) high‐density lipoprotein cholesterol (HDL‐C); (13) lower education level; (14) with cerebrovascular disease (CVD); (15) C‐reactive protein (CRP) >10 mg/L; (16) using insulin.

Abbreviations: F, sample size of females; M, sample size for males; T, total sample size; NA, not applicable.

^a^ Prevalence of malnutrition in patients with diabetes.

^b^ Prevalence of malnutrition and at‐risk for malnutrition in diabetics.

### 3.3 Quality assessment

All cohort and case–control studies included in this meta‐analysis underwent quality assessment using the Newcastle‐Ottawa Scale (NOS). Two articles were rated 9 points, five articles scored 8 points, two articles scored 7 points, and one article scored 6 points. These scores indicate that these studies were of high quality. The cross‐sectional studies were assessed using the JBI tool for cross‐sectional studies, with all studies achieving a quality score above 50%, and a mean score of 83%. Hence, they were considered to be of low risk in terms of methodological quality. A more detailed account of the methodological quality assessment is provided in the supplemental material (Tables S3 and S4).

### 3.4 Prevalence of malnutrition for patients with diabetes

**FIGURE 2:**
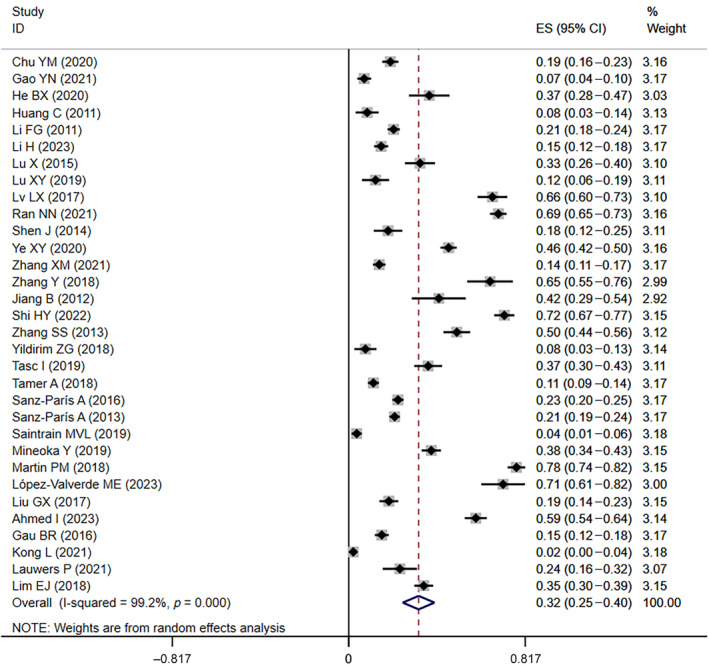
The forest plot of prevalence of malnutrition in diabetic patients. CI, confidence interval; ES, effect size.

In the 32 included studies^3^, ^4^, ^8^, ^10^, ^11^, ^12^, ^13^, ^14^, ^15^, ^19^, ^21^, ^22^, ^24^, ^25^, ^26^, ^27^, ^28^, ^29^, ^30^, ^34^, ^36^, ^37^, ^38^, ^40^, ^43^, ^44^, ^45^, ^46^, ^48^, ^50^, ^55^, ^56^ that provided data on the prevalence of malnutrition in diabetics, the prevalence of malnutrition ranged from 2% to 78%. The overall malnutrition prevalence was 33% (95% CI: 0.25–0.40, *I* ^*2*^ = 99.2%, *p* < 0.01) based on a random‐effects model meta‐analysis of all data, as shown in Figure 2.

**FIGURE 3:**
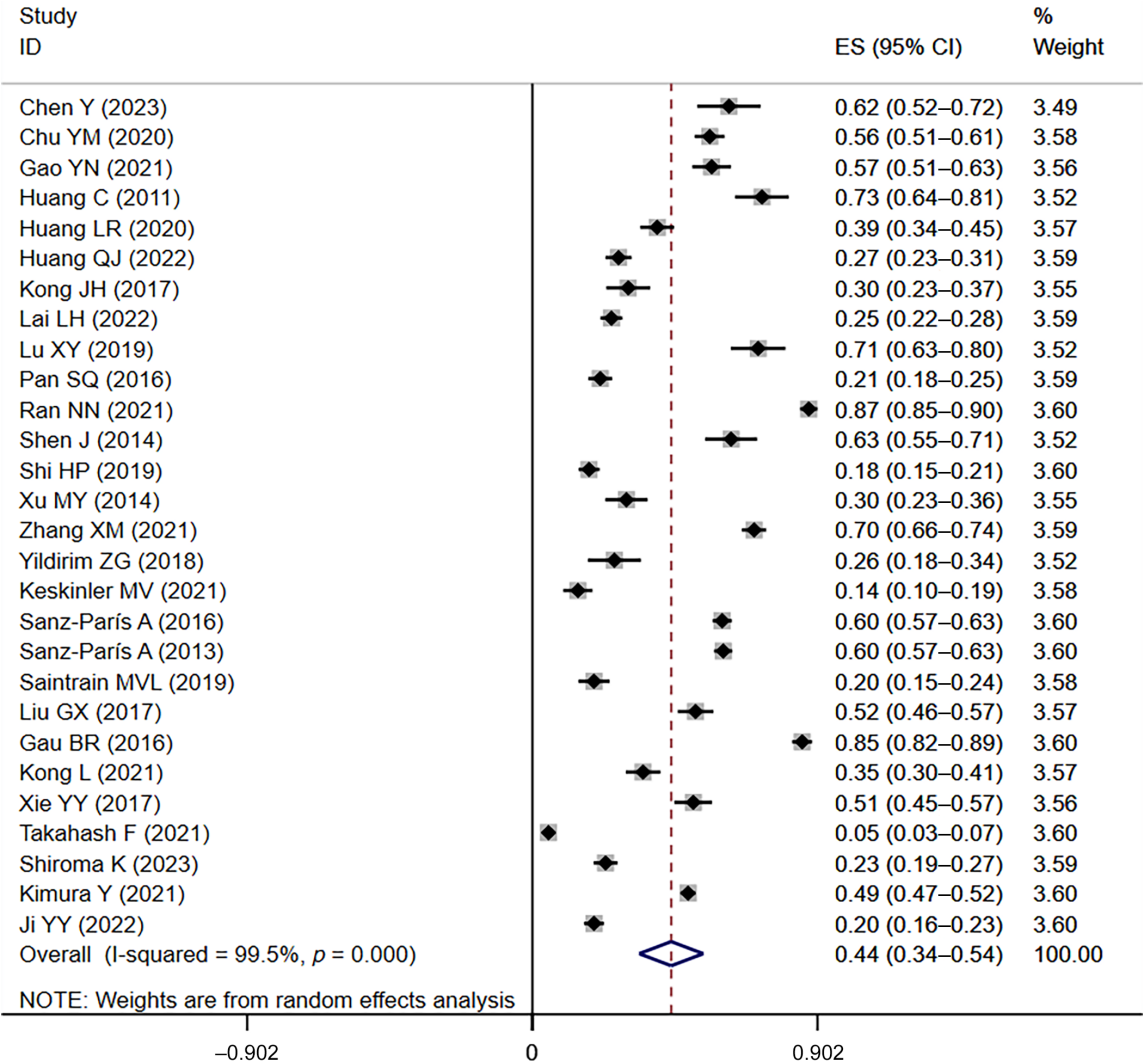
The forest plot of the prevalence of at‐risk for malnutrition in diabetic patients. CI, confidence interval; ES, effect size.

Similarly, in the 28 included studies^3^, ^10^, ^12^, ^17^, ^18^, ^20^, ^22^, ^23^, ^26^, ^27^, ^30^, ^34^, ^35^, ^37^, ^39^, ^40^, ^41^, ^42^, ^44^, ^47^, ^48^, ^49^, ^50^, ^51^, ^52^, ^53^, ^54^, ^55^ that provided data on the prevalence of at‐risk for malnutrition in diabetics, the prevalence of at‐risk for malnutrition ranged from 5% to 87%. The overall prevalence of at‐risk for malnutrition was 44% (95% CI: 0.34–0.54, *I* ^*2*^ = 99.5%, *p* < 0.01) based on a random‐effects model meta‐analysis of all data, as shown in Figure 3.

### 3.5 Subgroup analysis

Subgroup analyses were conducted on categorical variables, including the measurement tool, chronic complications of diabetes, and country of study, in order to explore differences in the prevalence of malnutrition across studies. The highest prevalence of malnutrition (47%, 95% CI: 0.01–0.94) was observed when using the Global Leadership Initiative on Malnutrition (GLIM) tool, followed by 43% for Controlling Nutritional Status (CONUT), 40% for Subjective Global Assessment (SGA), 30% for MNA, and 24% for Short‐Form Mini Nutritional Assessment (MNA‐SF). Patients with diabetic chronic complications had the highest prevalence of malnutrition (49%, 95% CI: 0.31–0.66), compared with 27% in patients without diabetic chronic complications. Moreover, the prevalence of malnutrition was highest in Spain (50%, 95% CI: 0.24–0.75), followed by 31% in China, and 18% in Turkey. These differences were statistically significant. Pooled estimates from subgroup analyses are provided in Table 2.

**TABLE 2:** Subgroups analysis of malnutrition.

Subgroups		*N*	Prevalence	Malnutrition 95% CI	*I* ^*2*^ (%)	*p* value	*χ* ^*2*^	*p* value
Measurement tool	MNA	14	30%	17%–42%	99.4%	<0.01	204.57	<0.01
	MNA‐SF	6	24%	15%–34%	97.0%	<0.01		
	CONUT	5	43%	20%–66%	99.3%	<0.01		
	SGA	3	40%	12%–69%	99.1%	<0.01		
	GLIM	2	47%	1%–94%	98.1%	<0.01		
Chronic complications of diabetes	None	23	27%	20%–34%	99.0%	<0.01	480.46	<0.01
	Yes	9	49%	31%–66%	98.9%	<0.01		
Country	China	20	31%	22%–41%	99.2%	<0.01	81.00	<0.01
	Turkey	3	18%	4%–33%	96.5%	<0.01		
	Spain	4	50%	24%–75%	99.5%	<0.01		

Abbreviations: CI, confidence interval; CONUT, Controlling Nutritional Status; GLIM, Global Leadership Initiative on Malnutrition; MNA, Mini Nutritional Assessment; MNA‐SF, Short‐Form Mini Nutritional Assessment; *N*, number of included studies; SGA, Subjective Global Assessment.

Subgroup analyses were conducted using the same categorical variables as malnutrition to explore differences in the prevalence of at‐risk for malnutrition across studies. The highest prevalence of at‐risk for malnutrition (59%, 95% CI: 0.56–0.61) was observed when using the MNA‐SF tool, followed by 58% for MNA, 30% for Geriatric Nutritional Risk Index (GNRI), and 29% for Nutritional Risk Screening 2002 (NRS‐2002). Patients with diabetes chronic complications had an 8% higher prevalence of at‐risk for malnutrition (51%, 95% CI: 0.30–0.73) compared with patients without diabetes chronic complications (44%, 95% CI: 0.34–0.54). Moreover, the prevalence of at‐risk for malnutrition was highest in Spain (60%, 95% CI: 0.58–0.62), followed by 49% in China, 26% in Japan, and 20% in Turkey. These differences were statistically significant. Pooled estimates from subgroup analyses are provided in Table 3.

**TABLE 3:** Subgroups analysis of at‐risk for malnutrition.

Subgroups		*N*	Prevalence	At‐risk for malnutrition (95% CI)	*I* ^*2*^ (%)	*p* value	*χ* ^*2*^	*p* value
Measurement tool	MNA	10	58%	44%–72%	99.1%	<0.01	1300.00	<0.01
	MNA‐SF	4	59%	56%–61%	8.9%	0.35		
	GNRI	5	30%	10%–49%	99.6%	<0.01		
	NNRS‐2002	9	29%	23%–35%	93.7%	<0.01		
Chronic complications of diabetes	None	20	44%	34%–54%	99.3%	<0.01	161.12	<0.01
	Yes	8	51%	30%–73%	99.5%	<0.01		
Country	China	20	49%	36%–61%	99.3%	<0.01	81.00	<0.01
	Turkey	2	20%	8%–31%	80.2%	0.02		
	Spain	2	60%	58%–62%	0.0%	0.88		
	Japan	3	26%	4%–56%	99.8%	<0.01	377.65	<0.01

Abbreviations: CI, confidence interval; GNRI, Geriatric Nutritional Risk Index; MNA, Mini Nutritional Assessment; MNA‐SF, Short‐Form Mini Nutritional Assessment; *N*, number of included studies; NRS‐2002, Nutritional Risk Screening 2002.

### 3.6 Influence factors associated with malnutrition for patients with diabetes

This meta‐analysis identified 16 potential influencing factors associated with malnutrition in diabetic patients, and the results are presented in Table 4. Diabetics with malnutrition had significantly lower levels of BMI, ALB, hemoglobin (Hb), TG, and high‐density lipoprotein cholesterol (HDL‐C), while showing an increase in glycosylated Hb (HbA1c). Furthermore, CRP >10 mg/L, age, duration of diabetes, Wagner grades 3–5, with cardiovascular disease (CVD), and using insulin were identified as risk factors for malnutrition in diabetic patients. There is insufficient evidence to establish an association between four other factors, namely smoking, gender, diabetic foot infection, and lower education level, with malnutrition in diabetic patients.

**TABLE 4:** Analyses of the influence factors of malnutrition in diabetic patients.

No.	Influence factors	Number of included studies	Heterogeneity		*I* ^*2*^	*p* value
			OR*/MD	95% CI		
1	BMI, kg/m^2^	12	−2.89	[−3.69 to −2.10]	91.58%	<0.01
2	ALB, g/L	11	−5.18	[−6.26 to −4.09]	90.78%	<0.01
3	Hb, g/L	7	−11.28	[−15.09 to −7.47]	84.19%	<0.01
4	TG, mmol/L	5	−0.42	[−0.65 to −0.18]	88.71%	<0.01
5	HDL‐C, mmol/L	3	−0.14	[−0.20 to −0.09]	67.36%	<0.01
6	HbA1c, %	10	1.06	[0.50 to 1.61]	94.47%	<0.01
7	CRP >10 mg/L	3	2.1*	[1.33 to 3.31]	53.2%	<0.01
8	Age, years	9	4.92	[3.39 to 6.46]	65.63%	<0.01
9	Duration of diabetes, years	6	2.97	[2.11 to 3.82]	53.88%	<0.01
10	Wagner grades 3–5	3	8.34*	[5.02 to 13.86]	0.0%	<0.01
11	With CVD	3	1.91*	[1.25 to 2.91]	0.0%	<0.01
12	Using insulin	3	1.75*	[1.09 to 2.80]	69.4%	0.02
13	Smoking	2	1.17*	[0.26 to 5.23]	90.5%	0.84
14	Female sex	5	1.11*	[0.70 to 1.74]	71.8%	0.66
15	With diabetic foot infection	2	3.06*	[0.84 to 11.13]	81.8%	0.09
16	Lower education level	3	2.40*	[0.87 to 6.64]	64.5%	0.09

Abbreviations: ALB, albumin; BMI, body mass index; CI, confidence interval; CRP, C‐reactive protein; CVD, cerebrovascular disease; Hb, hemoglobin; HbA1c, glycosylated hemoglobin; HDL‐C, high‐density lipoprotein cholesterol; MD, mean difference; OR*, odds ratio; TG, triglyceride.

### 3.7 Sensitivity analysis and tests for publication bias

**FIGURE 4:**
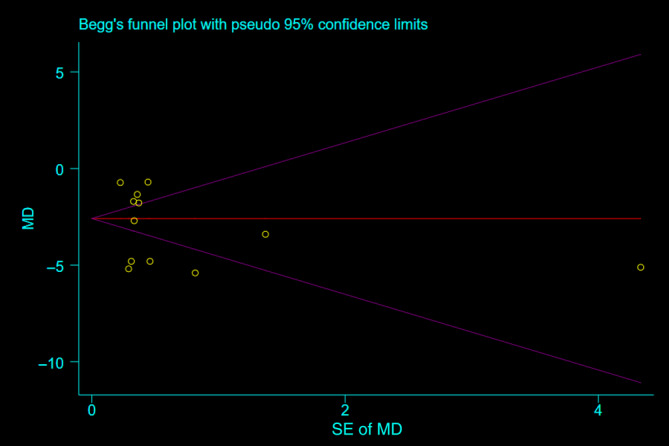
Funnel plot for assessing publication biases. MD, mean difference.

Sensitivity analysis of malnutrition prevalence and the prevalence of at‐risk for malnutrition was conducted by systematically excluding individual studies one by one, and the results showed that the comprehensive effect size did not change significantly before and after exclusion, indicating that the study results were relatively stable (Figures S1 and S2). To assess publication bias for factors that were examined in more than 10 articles (including BMI and ALB), Egger's test was performed. The results indicated the absence of publication bias. Taking BMI as an example, the funnel plot is presented in Figure 4, and the Egger's test showed a *p* value of 0.524, further confirming the absence of publication bias.

### 3.8 GRADE summary of findings

GRADE ratings for the relevant between influencing factors with malnutrition are provided in the supplemental material (Figure S3). GRADE quality of the evidence ranged from very low to high.

## 4 DISCUSSION

This meta‐analysis encompassed 46 studies comprising a total of 18 062 patients, aiming to investigate the prevalence of malnutrition and the influencing factors in diabetic patients. The overall prevalence of malnutrition in diabetic patients was found to be 33%, and the prevalence of at‐risk for malnutrition was 44%. We included and analyzed 16 influencing factors related to malnutrition in diabetic patients. Among these factors, BMI, ALB, Hb, TG, HDL‐C, HbA1c, CRP >10 mg/L, age, duration of diabetes, Wagner grades 3–5, and using insulin were significantly associated with malnutrition in diabetic patients.

Our subgroup analysis revealed variations in the prevalence of malnutrition and the prevalence of at‐risk for malnutrition across different countries. Specifically, the prevalence was found to be higher in Spain compared with China, higher in Japan compared to Turkey, and higher in developed countries compared to developing countries. These results differ from previous studies,^57^ which may be attributed to potential limitations introduced by sampling bias. One possible explanation for the discrepancy is that the prevalence of malnutrition was highest in the Spanish sample, but it also had the highest mean age, with a concentration between 69.6 and 80.8 years. Furthermore, it is plausible that despite more developed economies in certain countries, the prevalence of malnutrition remains elevated among older individuals with restricted mobility at home.^58^ Socioeconomic factors, access to healthcare, and cultural dietary practices could also play a role in contributing to the observed differences in malnutrition prevalence among different countries.

Diabetic patients with chronic complications had a 22% higher prevalence of malnutrition compared to those without complications. This association suggests that chronic complications of diabetes may contribute significantly to the increased prevalence of malnutrition in diabetic individuals.^56^ The presence of chronic complications is often characteristic of the later stages of diabetes, and patients with such complications tend to have a longer disease duration. Consequently, their bodies experience prolonged periods of high metabolism, heightened energy expenditure, and increased protein consumption, all of which elevate the risk of developing malnutrition.^59^ In the future, we should pay more attention to patients with chronic complications of diabetes.

The prevalence of malnutrition and at‐risk for malnutrition in diabetic patients is influenced by the measurement tools used. The lack of a standardized gold standard for diagnosing malnutrition leads to variations in results among different measurement tools.^60^ A meta‐analysis of 83 studies and 32 screening tools found that no single screening tool is universally applicable for all patients.^61^ Presently, the tools used to assess malnutrition in diabetic patients are diverse, and there is a lack of a specific screening tool tailored for malnourished diabetic individuals. Consider the adverse effects of malnutrition on the patient's prognosis, as well as the increase in treatment costs.^5^ There is an urgent need to develop a more reliable malnutrition screening tool to increase the identification of malnutrition.

The meta‐analysis results indicate that the malnutrition group had significantly lower BMI, ALB, and Hb values compared with the normal nutrition group. These differences were statistically significant, consistent with previous studies.^62^ BMI is widely used in existing malnutrition assessment tools and is recognized as a nutritional evaluation index. The World Health Organization (WHO) defines a BMI < 18.5 kg/m^2^ as malnutrition.^5^, ^63^ However, BMI is affected by whether the patient is overweight or obese in the past. For example, although the weight of the patient has been reduced by more than 20% within 3 months due to diseases (such as tuberculosis), the BMI value of the patient may still be normal or high due to obesity and other reasons.^64^ Similarly, ALB is commonly used as an evaluation indicator in some malnutrition assessments, but it can also decrease in the presence of inflammation, regardless of the patient's nutritional status.^65^, ^66^ One study^67^ pointed out that Hb is considered an indicator of malnutrition risk in the elderly, reflecting iron metabolism and protein status. However, a reduction in Hb levels only indicates the risk of malnutrition and may not accurately reflect the overall nutritional status of patients. Therefore, relying solely on these indicators may not provide a comprehensive and accurate assessment of the nutritional status of patients.

The malnutrition group showed significantly lower levels of TG and HDL‐C compared to the normal nutrition group. Reduced TG levels may hinder proper lipid and fat‐soluble vitamin absorption, increasing the risk of malnutrition. Additionally, low TG levels are closely related to inflammation, further elevating the risk of malnutrition.^51^ HDL‐C plays a crucial role in providing energy through metabolism. In cases of malnutrition‐induced high energy consumption, HDL‐C levels may decrease, affecting muscle mass and function.^68^ Patients with malnutrition also exhibited higher levels of HbA1c, which can disrupt glucose and amino acid metabolism, contributing to malnutrition in diabetic patients.^69^ CRP >10 mg/L emerged as a risk factor for malnutrition in diabetic patients. High CRP levels, indicative of systemic inflammatory response, can accelerate protein breakdown, leading to malnutrition.^70^

Comparing the mean age of diabetics with different nutritional status, the study results showed that the age of patients in the malnutrition group was significantly higher than that in the normal nutrition group. Aging is associated with a decline in enteric plexus nerve cells, affecting digestion and nutrient absorption, thereby increasing the risk of malnutrition.^71^ Furthermore, patients with malnutrition generally had a longer duration of diabetes than those with normal nutrition. As diabetes progresses, the declining function of pancreatic islets in diabetic patients can exacerbate protein consumption, further raising the risk of malnutrition.^25^

Patients with Wagner grades 3–5 are at a higher risk of malnutrition compared with those with Wagner grades 0–3, as higher Wagner grades indicate more severe infection, leading to increased protein consumption and higher susceptibility to malnutrition.^38^, ^72^ Patients with CVD often experience activated inflammatory responses, resulting in increased metabolic rates and higher protein breakdown and often cause digestive system issues, such as gastrointestinal bleeding and nausea, are common in CVD patients, causing reduced appetite and insufficient nutrient intake.^73^ Additionally, diabetic patients treated with insulin face strict dietary restrictions, including limited carbohydrate intake, which can result in inadequate nutrient intake and potential malnutrition.^74^

In contrast, our study found that smoking was not a risk factor for malnutrition in patients with diabetes. This is consistent with a previous meta‐analysis^75^ that investigated risk factors for malnutrition in patients with stroke. Although some studies have suggested that smoking may contribute to malnutrition and that smokers may have healthier diet compared with nonsmokers,^76^ the evidence on the impact of smoking on nutritional status in diabetic patients remains limited in this meta‐analysis. Additional research is required to generate more conclusive evidence in the future.

There is insufficient evidence to establish an association between three other factors, namely gender, diabetic foot infection, and lower education level, with malnutrition in diabetic patients. These factors might have been influenced by ethnicity, sample size, and the limited number of included studies. Further research is warranted to explore these factors comprehensively.

A study^77^ has shown that only 24.1% of diabetics exhibit good dietary compliance, and dietary compliance is usually closely related to the extent of dietary education provided by healthcare professionals. Moreover, comprehensive exercise training is beneficial in controlling patients' fasting blood glucose levels, enhancing muscle strength, improving physical function, and delaying the progression of frailty.^78^ Appropriate exercise training also promotes gastrointestinal activity, thereby increasing appetite and ensuring nutrient intake. Therefore, healthcare professionals should strengthen health education on dietary management for diabetics, provide effective health education, and develop reasonable dietary and exercise intervention plans to improve patients' nutritional status and prognosis.

This meta‐analysis possesses several strengths. First, it is the first systematic review and meta‐analysis to examine the prevalence of malnutrition and the prevalence of at‐risk for malnutrition, as well as the influencing factors of malnutrition in diabetic patients. Second, the included studies were of high quality, ensuring the reliability of the results. However, certain limitations should be acknowledged. First, the lack of a gold standard for malnutrition and at‐risk for malnutrition, along with the heterogeneity in measurement tools, may have affected data integration and, to some extent, the results. Furthermore, the number of studies included in the analysis of influencing factors was relatively small, limiting the strength of evidence‐based conclusions. Lastly, the search strategy was restricted to English and Chinese, which may have limited the comprehensiveness of the included studies. Given these limitations, the findings should be interpreted cautiously. Further in‐depth studies are necessary to validate and expand upon the outcomes of this study in the future.

## 5 CONCLUSION

Based on the results of our meta‐analysis, the prevalence of malnutrition and the prevalence of at‐risk for malnutrition in diabetics were 33% and 44%, respectively. The BMI, ALB, Hb, TG, HDL‐C, HbA1c, age, and duration of diabetes were significantly different between different nutritional status groups. Wagner grades 3–5, with CVD and using insulin may increase the risk of malnutrition in diabetic patients. Furthermore, based on our analysis, smoking is most likely not a risk factor for malnutrition in patients with diabetes. The effects of gender, with diabetic foot infection, and lower education level on malnutrition in diabetics are unclear. It is essential to conduct more studies in the future to enhance the reliability and validity of our findings. Given the negative impact of malnutrition on health outcomes, regular nutrition screening is recommended for patients with the identified risk factors. Early detection and treatment of malnutrition, and reduce the impact on individual health.

## AUTHOR CONTRIBUTIONS

All authors contributed to the study conception and design. Material preparation, data collection, and analysis were performed by Tong Zhang and Jiangxia Qin. The first draft of the manuscript was written by Tong Zhang, and all authors commented on previous versions of the manuscript. All authors read and approved the final manuscript. Lin Han handles correspondence at all stages of refereeing and publication, also post‐publication.

## FUNDING INFORMATION

This study was supported by National Nature Science Foundation of China (72274087, 71663002, 71704071), the fund of China Medical Board (#20‐374).

## CONFLICT OF INTEREST STATEMENT

The authors declare no conflicts of interest.

## Supporting information


**Figure S1.** Sensitivity analysis of malnutrition prevalence.
**Figure S2.** Sensitivity analysis of at‐risk for malnutrition prevalence.

**Figure S3.** GRADE ratings for the relevant between influencing factors with malnutrition.

**Table S1.** Search strategy.

**Table S2.** Measurement tools of nutritional disorder.

**Table S3.** Newcastle‐Ottawa Scale (NOS) assessment of the quality of cohort studies.
**Table S4.** Methodological quality assessment of included studies using the Joanna Briggs Institute's (JBI) critical appraisal checklist.
