# Supplementary material for: Prevalence and influencing factors of malnutrition in diabetic patients: A systematic review and meta‐analysis
Source: J Diabetes. 2024 Oct 4;16(10):e13610. doi: 10.1111/1753-0407.13610 (PMC11450603; doi:10.1111/1753-0407.13610)
Supplement: Supplementary file 1 — Figure S1. Sensitivity analysis of malnutrition prevalence. Figure S2. Sensitivity analysis of at‐risk for malnutrition prevalence. [file JDB-16-e13610-s001.docx]

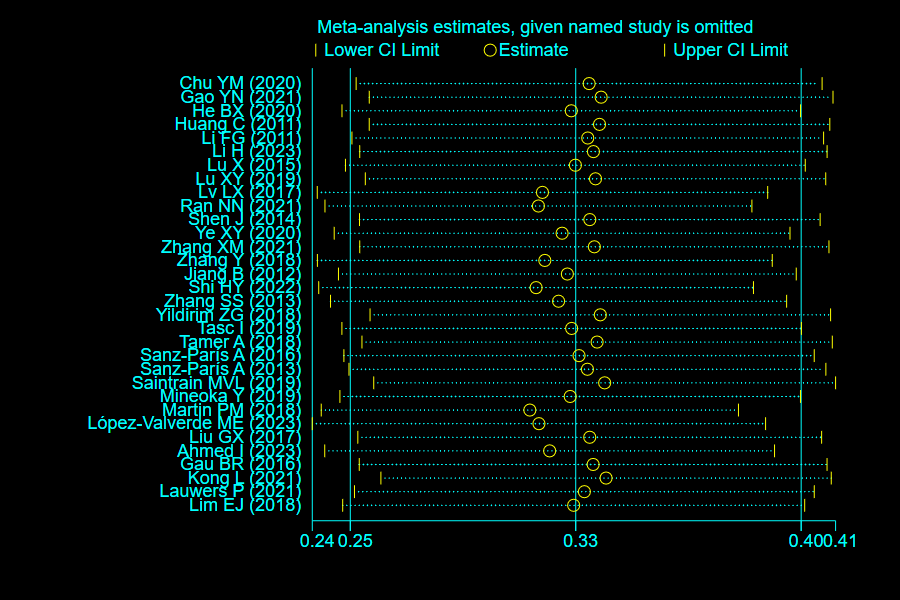


**Figure S1:** Sensitivity analysis of malnutrition prevalence


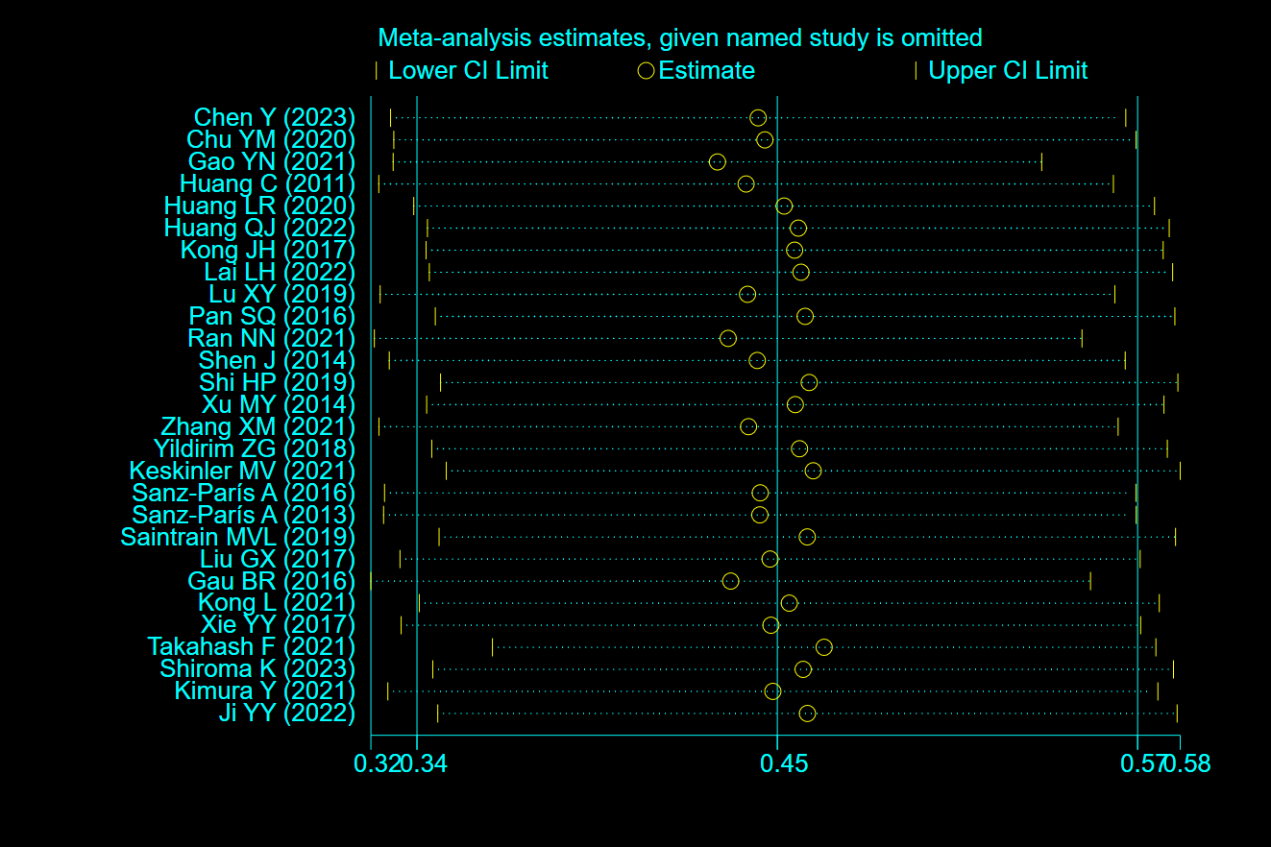


**Figure S2:** Sensitivity analysis of at-risk for malnutrition prevalence
