# Supplementary figures and images for: Prevalence and influencing factors of malnutrition in diabetic patients: A systematic review and meta‐analysis
Source: J Diabetes. 2024 Oct 4;16(10):e13610. doi: 10.1111/1753-0407.13610 (PMC11450603; doi:10.1111/1753-0407.13610)

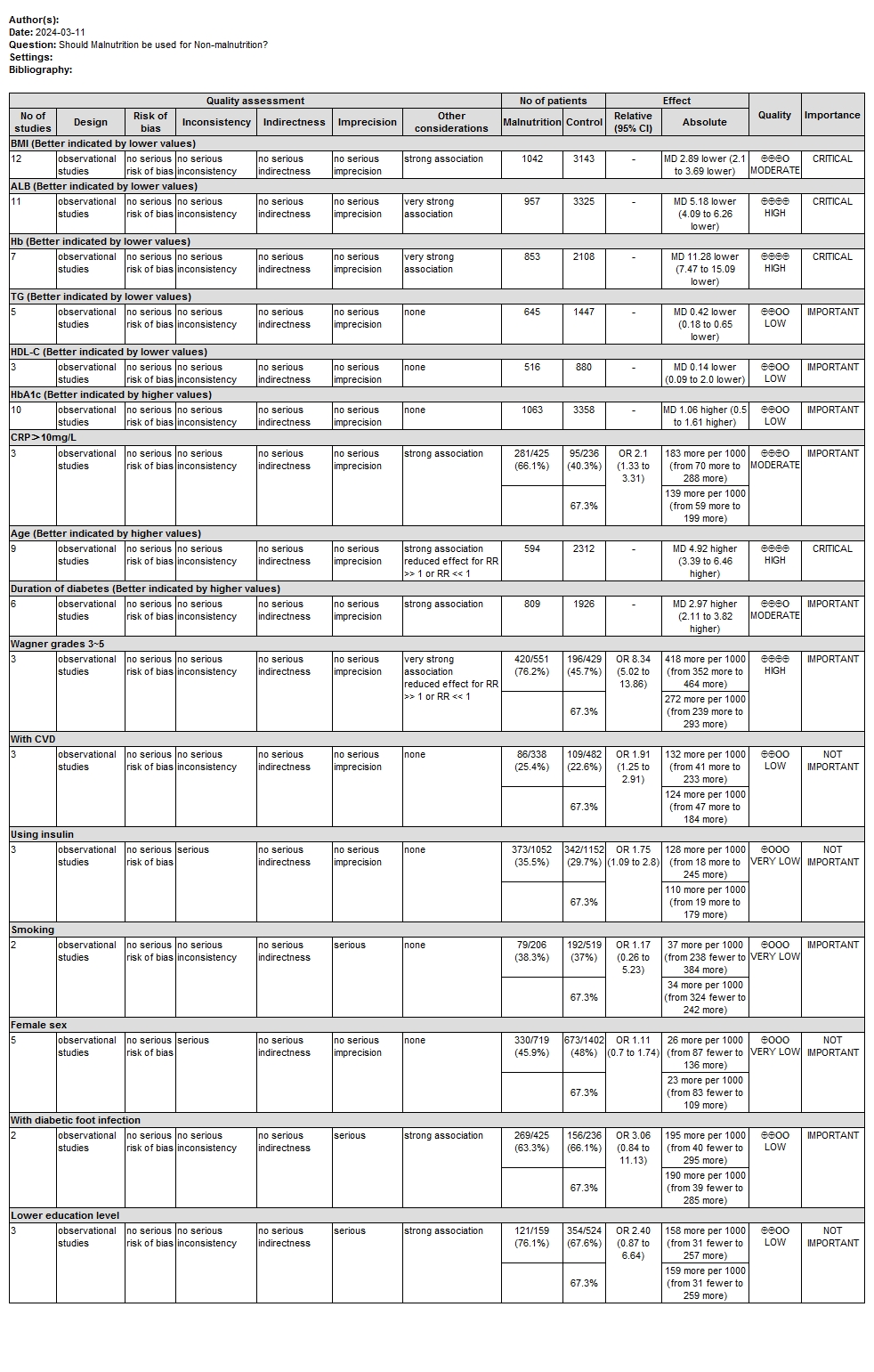


**Figure S3:** GRADE ratings for the relevant between influencing factors with malnutrition

Supplement: Supplementary file 2 — Figure S3. GRADE ratings for the relevant between influencing factors with malnutrition. [file JDB-16-e13610-s004.docx]
