# Supplementary material for: Prevalence and influencing factors of malnutrition in diabetic patients: A systematic review and meta‐analysis
Source: J Diabetes. 2024 Oct 4;16(10):e13610. doi: 10.1111/1753-0407.13610 (PMC11450603; doi:10.1111/1753-0407.13610)
Supplement: Supplementary file 3 — Table S1. Search strategy. [file JDB-16-e13610-s005.docx]

|  | **Wanfang** | Results |
| --- | --- | --- |
| #1 | 糖尿病（题名或关键词） OR 2型糖尿病（题名或关键词） OR 1型糖尿病（题名或关键词） |  |
| #2 | 营养不良（题名或关键词） OR 营养失调（题名或关键词） OR 营养下降（题名或关键词） OR 营养障碍（题名或关键词） OR 营养缺乏（题名或关键词） OR 营养不良风险（题名或关键词） OR 营养风险（题名或关键词） OR 营养状况（题名或关键词） OR 营养状态（题名或关键词） |  |
| #3 | 危险因素（题名或关键词） OR 影响因素（题名或关键词） OR 相关因素（题名或关键词） OR 高危因素（题名或关键词） OR 患病率（题名或关键词） OR 发病率（题名或关键词） OR 发生率（题名或关键词） OR 流行病学（题名或关键词） |  |
| #4 | **#1 AND #2 AND #3** | 250 |

**Supplementary Table S1 Search strategy**

|  | **CNKI** | Results |
| --- | --- | --- |
| #1 | 糖尿病（主题） + 2型糖尿病（主题） + 1型糖尿病（主题） |  |
| #2 | 营养不良（主题） + 营养失调（主题） + 营养下降（主题） + 营养障碍（主题） + 营养缺乏（主题） + 营养不良风险（主题） + 营养风险（主题） + 营养状况（主题） + 营养状态（主题） |  |
| #3 | 危险因素（主题） + 影响因素（主题） + 相关因素（主题） + 高危因素（主题） + 患病率（主题） + 发病率（主题） + 发生率（主题） + 流行病学（主题） |  |
| #4 | **#1 AND #2 AND #3** | 641 |

|  | **VIP** | Results |
| --- | --- | --- |
| #1 | 糖尿病（题名或关键词） OR 2型糖尿病（题名或关键词） OR 1型糖尿病（题名或关键词） |  |
| #2 | 营养不良（题名或关键词） OR 营养失调（题名或关键词） OR 营养下降（题名或关键词） OR 营养障碍（题名或关键词） OR 营养缺乏（题名或关键词） OR 营养不良风险（题名或关键词） OR 营养风险（题名或关键词） OR 营养状况（题名或关键词） OR 营养状态（题名或关键词） |  |
| #3 | 危险因素（题名或关键词） OR 影响因素（题名或关键词） OR 相关因素（题名或关键词） OR 高危因素（题名或关键词） OR 患病率（题名或关键词） OR 发病率（题名或关键词） OR 发生率（题名或关键词） OR 流行病学（题名或关键词） |  |
| #4 | **#1 AND #2 AND #3** | 85 |

|  | **Pubmed** | Results |
| --- | --- | --- |
| #1 | "Diabetes Mellitus"[Mesh] OR "Diabetes Mellitus"[Title/Abstract] OR Diabetes[Title/Abstract] OR Diabetic[Title/Abstract] OR "type 2 diabetes"[Title/Abstract] OR T2DM[Title/Abstract] OR Diabet*[Title/Abstract] OR "type 1 diabetes"[Title/Abstract] OR T1DM[Title/Abstract] |  |
| #2 | Malnutrition[Mesh] OR "malnutrition"[Title/Abstract] OR "nutritional deficiency"[Title/Abstract] OR "nutritional assessment"[Title/Abstract] OR "nutritional disorders"[Title/Abstract] OR "nutritional status"[Title/Abstract] OR "malnutrition screening"[Title/Abstract] OR "innutrition"[Title/Abstract] OR "nutritional risk"[Title/Abstract] |  |
| #3 | "Risk factors"[Mesh] OR "Risk factors"[Title/Abstract] OR "influence factors"[Title/Abstract] OR "associated factors"[Title/Abstract] OR "dangerous factors"[Title/Abstract] OR Incidence[Mesh] OR Incidence[Title/Abstract] OR Prevalence[Title/Abstract] OR Epidemiology[Mesh] OR Epidemiology[Title/Abstract] OR Frequency[Title/Abstract] |  |
| #4 | **#1 AND #2 AND #3** | 4038 |

|  | **CBM** | Results |
| --- | --- | --- |
| #1 | 糖尿病[核心字段] OR 2型糖尿病[核心字段] OR 1型糖尿病[核心字段] |  |
| #2 | 营养不良[核心字段] OR 营养失调[核心字段] OR 营养下降[核心字段] OR 营养障碍[核心字段] OR 营养缺乏[核心字段] OR 营养不良风险[核心字段] OR 营养风险[核心字段] OR 营养状况[核心字段] OR 营养状态[核心字段] |  |
| #3 | 患病率[核心字段] OR 发病率[核心字段] OR 发生率[核心字段] OR 流行病学[核心字段] OR 影响因素[核心字段] OR 危险因素[核心字段] OR 相关因素[核心字段] OR 高危因素[核心字段] |  |
| #4 | **#1 AND #2 AND #3** | 267 |

|  | **Cochrane Library** | Results |
| --- | --- | --- |
| #1 | MeSH[Diabetes Mellitus] OR ("Diabetes Mellitus" OR Diabetes OR Diabetic OR "type 2 diabetes" OR T2DM OR Diabet* OR "type 1 diabetes" OR T1DM):ti,ab,kw |  |
| #2 | MeSH [Malnutrition] OR ("Malnutrition" OR "nutritional deficiency" OR "nutritional assessment" OR "nutritional disorders" OR "nutritional status" OR "malnutrition screening" OR "innutrition" OR "nutritional risk"):ti,ab,kw |  |
| #3 | MeSH [Risk Factors] OR ("Risk factors" OR "influence factors" OR "associated factors" OR "dangerous factors" OR Incidence OR Prevalence OR Epidemiology OR Frequency):ti,ab,kw |  |
| #4 | **#1 AND #2 AND #3** | 728 |

|  | **Embase** | Results |
| --- | --- | --- |
| #1 | ‘Diabetes Mellitus’:ab,ti OR Diabetes:ab,ti OR Diabetic:ab,ti OR ‘type 2 diabetes’:ab,ti OR T2DM:ab,ti OR Diabet*:ab,ti OR ‘type 1 diabetes’:ab,ti OR T1DM:ab,ti |  |
| #2 | ‘Malnutrition’:ab,ti OR ‘nutritional deficiency’:ab,ti OR ‘nutritional assessment’:ab,ti OR ‘nutritional disorders’:ab,ti OR ‘nutritional status’:ab,ti OR ‘malnutrition screening’:ab,ti OR ‘innutrition’:ab,ti OR ‘nutritional risk’:ab,ti |  |
| #3 | ‘Risk factors’:ab,ti OR ‘influence factors’:ab,ti OR ‘associated factors’:ab,ti OR ‘dangerous factors’:ab,ti OR Incidence/exp OR Incidence:ab,ti OR Prevalence:ab,ti OR Epidemiology:ab,ti OR Frequency:ab,ti |  |
| #4 | **#1 AND #2 AND #3** | 3137 |

|  | **Web of science** | Results |
| --- | --- | --- |
| #1 | TS=("Diabetes Mellitus" OR Diabetes OR Diabetic OR "type 2 diabetes" OR T2DM OR Diabet* OR "type 1 diabetes" OR T1DM) |  |
| #2 | TS=(Malnutrition OR "nutritional deficiency" OR "nutritional assessment" OR "nutritional disorders" OR "nutritional status" OR "malnutrition screening" OR "innutrition" OR "nutritional risk") |  |
| #3 | TS=("Risk factors" OR "influence factors" OR "associated factors" OR "dangerous factors" OR Incidence OR Prevalence OR Epidemiology OR Frequency) |  |
| #4 | **#1 AND #2 AND #3** | 2328 |
