# Supplementary material for: Prevalence and influencing factors of malnutrition in diabetic patients: A systematic review and meta‐analysis
Source: J Diabetes. 2024 Oct 4;16(10):e13610. doi: 10.1111/1753-0407.13610 (PMC11450603; doi:10.1111/1753-0407.13610)
Supplement: Supplementary file 4 — Table S2. Measurement tools of nutritional disorder. [file JDB-16-e13610-s003.docx]

**Supplementary Table S2 Measurement tools of nutritional disorder**

| **Measurement tools of malnutrition** | |
| --- | --- |
| SGA^[1-3]^ | Malnutrition: rating B and C |
| NRI^[4]^ | Malnutrition: ≤100 points |
| MNA^[1-3]^ | Malnourished：＜17 points |
| MNA-SF^[5]^ | Malnourished：0 – 7 points |
| GLIM ^[6]^ | A two-step approach for the malnutrition diagnosis was selected.  First phase: screening to identify “at risk” status by the use of any validated screening tool.  Second phase: required at least one phenotypic criterion (Non-volitional weight loss; Low body mass index; Reduced muscle mass) and one etiologic criterion (Reduced food intake or assimilation; Inflammation) |
| COUNT^[7]^ | Normal nutrition: 0 ~ 1 points  Mild malnutrition: 2 to 4 points  Moderate malnutrition: 5 to 8 points  Severe malnutrition: 9 to 12 points |
| NSI ^[8]^ | Malnutrition: ≥6 points |
| **Measurement tools of at-risk for malnutrition** | |
| MNA^[9,10]^ | At risk of malnutrition/malnutrition:＜24 points |
| MNA-SF^[5]^ | At risk of malnutrition/malnutrition: ≤11 points |
| NRS 2002^[11]^ | Nutritionally at-risk：≥3 points |
| GNRI^[12]^ | Severe risk： <82 score points  Moderate risk：82 to 92 points  Low risk：92 to 98 points  No risk：>98 points |

MNA: Mini Nutritional Assessment; MNA-SF: Short-Form Mini-Nutritional Assessment; SGA: Subjective Global Assessment; NRI: Nutritional Risk Index GLIM: Global Leadership Initiative on Malnutrition; COUNT: Controlling Nutritional Status; NSI: Nutritional Screening Initiative; NRS-2002: Nutritional Risk Screening 2002; GNRI: Geriatric Nutritional Risk Index.

**References**

1 Marshall, S., Young, A., Bauer, J. *et al.* Malnutrition in Geriatric Rehabilitation: Prevalence, Patient Outcomes, and Criterion Validity of the Scored Patient-Generated Subjective Global Assessment and the Mini Nutritional Assessment. *J Acad Nutr Diet* **116**, 785-794 (2016), doi:10.1016/j.jand.2015.06.013

2 Crichton, M., Craven, D., Mackay, H. *et al.* A systematic review, meta-analysis and meta-regression of the prevalence of protein-energy malnutrition: associations with geographical region and sex. *Age and ageing* **48**, 38-48 (2019), doi:10.1093/ageing/afy144

3 Marshall, S., Craven, D., Kelly, J. *et al.* A systematic review and meta-analysis of the criterion validity of nutrition assessment tools for diagnosing protein-energy malnutrition in the older community setting (the MACRo study). *Clin Nutr* **37**, 1902-1912 (2018), doi:10.1016/j.clnu.2017.09.022

4 Poulia, K. A., Yannakoulia, M., Karageorgou, D. *et al.* Evaluation of the efficacy of six nutritional screening tools to predict malnutrition in the elderly. *Clinical nutrition (Edinburgh, Scotland)* **31**, 378-385 (2012), doi:10.1016/j.clnu.2011.11.017

5 Kaiser, M. J., Bauer, J. M., Ramsch, C. *et al.* Validation of the Mini Nutritional Assessment short-form (MNA-SF): a practical tool for identification of nutritional status. *J Nutr Health Aging* **13**, 782-788 (2009), doi:10.1007/s12603-009-0214-7

6 Jensen, G. L., Cederholm, T., Correia, M. *et al.* GLIM Criteria for the Diagnosis of Malnutrition: A Consensus Report From the Global Clinical Nutrition Community. *JPEN J Parenter Enteral Nutr* **43**, 32-40 (2019), doi:10.1002/jpen.1440

7 Ignacio de Ulíbarri, J., González-Madroño, A., de Villar, N. G. *et al.* CONUT: a tool for controlling nutritional status. First validation in a hospital population. *Nutricion hospitalaria* **20**, 38-45 (2005)

8 Lim, E. J. Factors Influencing Mobility Relative to Nutritional Status among Elderly Women with Diabetes Mellitus. *Iranian journal of public health* **47**, 814-823 (2018)

9 Fu, J., Li, Z., Wang, F. *et al.* Prevalence of malnutrition/malnutrition risk and nutrition-related risk factors among patients with Parkinson's disease: systematic review and meta-analysis. *Nutr Neurosci*, 1-11 (2021), doi:10.1080/1028415X.2021.1948655

10 Visvanathan, R., Penhall, R. & Chapman, I. Nutritional screening of older people in a sub-acute care facility in Australia and its relation to discharge outcomes. *Age Ageing* **33**, 260-265 (2004), doi:10.1093/ageing/afh078

11 Kondrup, J. ESPEN Guidelines for Nutrition Screening 2002. *Clinical Nutrition* **22**, 415-421 (2003), doi:10.1016/s0261-5614(03)00098-0

12 Bouillanne, O., Morineau, G., Dupont, C. *et al.* Geriatric Nutritional Risk Index: a new index for evaluating at-risk elderly medical patients. *The American journal of clinical nutrition* **82**, 777-783 (2005), doi:10.1093/ajcn/82.4.777
