# Supplementary material for: Prevalence and influencing factors of malnutrition in diabetic patients: A systematic review and meta‐analysis
Source: J Diabetes. 2024 Oct 4;16(10):e13610. doi: 10.1111/1753-0407.13610 (PMC11450603; doi:10.1111/1753-0407.13610)
Supplement: Supplementary file 5 — Table S3. Newcastle‐Ottawa Scale (NOS) assessment of the quality of cohort studies. Table S4. Methodological quality assessment of included studies using the Joanna Briggs Institute's (JBI) critical appraisal checklist. [file JDB-16-e13610-s002.docx]

**Table S3 Newcastle-Ottawa Scale (NOS) assessment of the quality of cohort studies**

| Cohort  studies  score | Selection | | | | Comparability Comparable | Outcome | | | |
| --- | --- | --- | --- | --- | --- | --- | --- | --- | --- |
|  | Exposed cohort | Non-exposed cohort | Ascertainment of exposure | Outcome  of interest |  | Assessment  of outcome | Length of follow-up | Adequacy of follow-up | Total |
| Huang LR (2020) | * | * | * | * | ** | * | - | * | 8 |
| Zhang SS (2013) | * | * | * | * | ** | * | - | * | 8 |
| Xie YY (2017) | * | * | * | * | ** | * | * | * | 9 |
| Sanz-París A (2016) | * | * | * | * | ** | * | - | * | 8 |
| Sanz-París A (2013) | * | * | * | * | * | * | - | * | 7 |
| Martin PM (2018) | * | * | * | * | * | * | - | * | 7 |
| López-Valverde ME  (2023) | * | * | * | * | * | * | * | * | 8 |
| Liu GX (2017) | * | * | * | * | ** | * | * | * | 9 |
| Kimura Y (2021) | * | * | * | * | ** | * | - | * | 8 |
| Lauwers P (2021) | * | * | * | - | * | * | - | * | 6 |

The methodological quality assessment of the case-control/cohort study was in accordance with the Newcastle-Ottawa Scale (NOS, studies with a score≥6 were considered as the high quality.).

**Table S4 Methodological quality assessment of included studies using the Joanna Briggs Institute’s (JBI) critical appraisal checklist**

| Study | Q1 | Q2 | Q3 | Q4 | Q5 | Q6 | Q7 | Q8 | Scores Yes | %Yes |
| --- | --- | --- | --- | --- | --- | --- | --- | --- | --- | --- |
| He BX (2020) | Yes | No | Yes | Yes | Yes | Yes | Yes | Yes | 7/8 | 88% |
| Huang C (2011) | Yes | No | Yes | Yes | No | No | Yes | No | 4/8 | 50% |
| Kong JH (2017) | Yes | No | Yes | Yes | No | No | Yes | No | 4/8 | 50% |
| Lu X (2015) | Yes | Yes | Yes | Yes | Yes | Yes | Yes | Yes | 8/8 | 100% |
| Lu XY (2019) | Yes | Yes | Yes | Yes | Yes | No | Yes | Yes | 7/8 | 88% |
| Lv LX (2017) | Yes | Yes | Yes | Yes | Yes | No | Yes | Yes | 7/8 | 88% |
| Pan SQ (2016) | Yes | Yes | Yes | Yes | Yes | No | Yes | Yes | 7/8 | 88% |
| Ran NN (2021) | Yes | Yes | Yes | Yes | Yes | Yes | Yes | Yes | 8/8 | 100% |
| Shen J (2014) | No | Yes | Yes | Yes | No | No | Yes | Yes | 5/8 | 63% |
| Shi HP (2019) | Yes | Yes | Yes | Yes | Yes | Yes | Yes | No | 7/8 | 88% |
| Xu MY (2014) | Yes | No | Yes | Yes | Yes | No | Yes | Yes | 6/8 | 75% |
| Zhang Y (2018) | Yes | No | Yes | Yes | Yes | Yes | Yes | Yes | 7/8 | 88% |
| Yildirim ZG (2018) | Yes | Yes | Yes | Yes | Yes | No | Yes | No | 6/8 | 75% |
| Keskinler MV (2021) | Yes | Yes | Yes | Yes | Yes | No | Yes | Yes | 7/8 | 88% |
| Tasc I (2019) | Yes | Yes | Yes | Yes | Yes | Yes | Yes | Yes | 8/8 | 100% |
| Tamer A (2018) | Yes | Yes | Yes | Yes | Yes | Yes | Yes | Yes | 8/8 | 100% |
| Takahash F (2021) | Yes | Yes | Yes | Yes | Yes | Yes | Yes | Yes | 8/8 | 100% |
| Shiroma K (2023) | Yes | Yes | Yes | Yes | Yes | Yes | Yes | Yes | 8/8 | 100% |
| Saintrain MV (2019） | Yes | Yes | Yes | Yes | Yes | No | Yes | Yes | 7/8 | 88% |
| Mineoka Y (2019) | Yes | Yes | Yes | Yes | Yes | No | Yes | Yes | 7/8 | 88% |
| Ji YY (2022) | Yes | Yes | Yes | Yes | Yes | Yes | Yes | No | 7/8 | 88% |
| Ahmed I (2023) | Yes | Yes | Yes | Yes | Yes | Yes | Yes | Yes | 8/8 | 100% |
| Gau BR (2016） | Yes | Yes | Yes | Yes | Yes | No | Yes | No | 6/8 | 75% |
| Kong L (2021) | Yes | Yes | Yes | Yes | Yes | Yes | Yes | No | 7/8 | 88% |
| Lim EJ (2018） | Yes | Yes | Yes | Yes | Yes | No | Yes | Yes | 7/8 | 88% |
| Chen Y (2023) | Yes | No | Yes | Yes | Yes | Yes | Yes | No | 6/8 | 75% |
| Chu YM (2020) | No | No | Yes | Yes | Yes | Yes | Yes | Yes | 6/8 | 75% |
| Gao YN (2021) | Yes | No | Yes | Yes | Yes | Yes | Yes | No | 6/8 | 75% |
| Huang QJ (2022) | Yes | No | Yes | Yes | Yes | Yes | Yes | Yes | 7/8 | 88% |
| Lai LH (2022) | Yes | Yes | Yes | Yes | Yes | Yes | Yes | Yes | 8/8 | 100% |
| Li FG (2011) | No | Yes | Yes | Yes | No | No | Yes | No | 4/8 | 50% |
| Li H (2023) | Yes | No | Yes | Yes | Yes | Yes | Yes | Yes | 7/8 | 88% |
| Ye XY (2020) | Yes | No | Yes | Yes | Yes | Yes | Yes | Yes | 7/8 | 88% |
| Zhang XM (2021) | Yes | Yes | Yes | Yes | No | No | Yes | No | 5/8 | 63% |
| Jiang B (2012) | No | No | Yes | Yes | Yes | No | Yes | No | 4/8 | 50% |
| Shi HY (2022) | Yes | Yes | Yes | Yes | Yes | Yes | Yes | Yes | 8/8 | 100% |

NOTE: Q1. Were the criteria for inclusion in the sample clearly defined? Q2. Were the study subjects and the setting described in detail? Q3. Was the exposure measured in a valid and reliable way? Q4. Were objective, standard criteria used for measurement of the condition? Q5. Were confounding factors identified? Q6. Were strategies to deal with confounding factors stated? Q7. Were the outcomes measured in a valid and reliable way? Q8. Was appropriate statistical analysis used? Studies that had 50% or more ‘Yes’ across the quality assessment parameters were considered low risk.
